# Lactate Dehydrogenase-to-Albumin Ratio and Glasgow Prognostic Score as Prognostic Markers in Metastatic Breast Cancer Treated with CDK4/6 Inhibitors

**DOI:** 10.3390/curroncol33100582

**Published:** 2026-09-29

**Authors:** Ece Sahin Hafizoglu, Ferhat Ekinci, Mustafa Sahbazlar, Atike Pinar Erdogan

**Affiliations:** Division of Medical Oncology, Faculty of Medicine, Celal Bayar University, 45030 Manisa, Turkey; ece.hafizoglu@cbu.edu.tr (E.S.H.); ekinci.ferhat@cbu.edu.tr (F.E.); mustafa.sahbazlar@cbu.edu.tr (M.S.)

**Keywords:** breast cancer, cyclin-dependent kinase 4/6 inhibitors, lactate dehydrogenase-to-albumin ratio, Glasgow Prognostic Score, prognosis

## Abstract

**Background:** CDK4/6 inhibitors with endocrine therapy are standard first-line treatment for hormone receptor-positive, HER2-negative metastatic breast cancer, yet simple pretreatment prognostic markers are lacking. The LDH-to-albumin ratio (LAR) and the Glasgow Prognostic Score (GPS) both incorporate serum albumin and are each reported as prognostic, but have not been compared within a single cohort, and LAR has not been examined during CDK4/6 inhibitor therapy. This study evaluated both indices in this setting. **Methods:** Consecutive patients (*n* = 139) receiving palbociclib or ribociclib plus endocrine therapy (2020–2025) were analysed retrospectively. LAR was modelled continuously on the logarithmic scale, with hazard ratios per doubling; an external cut-off (46.27) served for Kaplan–Meier illustration only. GPS was calculated in its original form. As both contain albumin, each entered a separate multivariable Cox model with identical covariates. Endpoints were overall survival (OS) and progression-free survival (PFS). **Results:** Over a median follow-up of 50.0 months, 72 deaths and 110 progression events occurred. LAR was independently associated with OS (adjusted HR 2.87 per doubling, 95% CI 1.78–4.62, *p* < 0.001) and PFS (2.21, 1.45–3.35, *p* < 0.001). GPS was independently associated with OS (1.72, 1.05–2.82, *p* = 0.032) but not PFS (1.32, 0.88–1.98, *p* = 0.187). LAR-based models had lower Akaike information criterion values (ΔAIC 12.3 and 10.9), but concordance did not differ significantly between the indices (ΔC ≈ 0.04, *p* > 0.20). **Conclusions:** Baseline LAR, an inexpensive routine index, was associated with both endpoints, whereas GPS was associated with overall survival alone. Discrimination was modest for both indices (C-index ≤ 0.61) and did not differ significantly between them. External validation is required.

## Simple Summary

Drugs called cyclin-dependent kinase 4/6 inhibitors, given with hormone therapy, are the standard first treatment for the most common type of advanced breast cancer, yet the disease of some patients worsens within months, whereas others remain on treatment for years. Blood tests reflecting tumour activity and the body’s inflammatory state have been linked to outcomes in other cancers. In 139 patients, a higher ratio of lactate dehydrogenase, an enzyme released by rapidly growing tumours, to albumin, a blood protein that falls with inflammation and poor nutrition, was linked to shorter survival and earlier worsening of the disease, whereas a score combining C-reactive protein and albumin was linked to survival only. Neither was accurate enough to estimate individual patients’ outcomes. If confirmed in other patient groups, this inexpensive ratio could help researchers identify higher-risk patients for further study and could be tested as an early signal of treatment response.

## 1. Introduction

Breast cancer is the most frequently diagnosed malignancy among women worldwide, accounting for approximately 2.3 million new cases and 666,000 deaths in 2022 [1]. Treatment is guided principally by hormone receptor (HR) and human epidermal growth factor receptor 2 (HER2) status, and HR-positive, HER2-negative disease constitutes the largest subgroup [2]. The importance of this classification extends across all disease stages; in HER2-positive early breast cancer, for instance, the neoadjuvant regimen selected has been shown to influence the outcomes subsequently achieved with adjuvant therapy [3]. For patients with HR-positive, HER2-negative metastatic disease, endocrine therapy combined with a cyclin-dependent kinase 4/6 (CDK4/6) inhibitor is established as the standard first-line approach [4].

CDK4/6 inhibitors arrest tumour proliferation by blocking the G1–S transition of the cell cycle and, being considerably more selective than earlier CDK inhibitors, are well tolerated in combination with endocrine therapy [5,6]. In randomised phase III trials, the addition of a CDK4/6 inhibitor to endocrine therapy substantially prolonged progression-free survival [7], and ribociclib has further demonstrated a significant overall survival benefit in both the first-line and the endocrine-pretreated setting [8,9]. The benefit is not uniform, however. An overall survival advantage has not been consistently demonstrated for palbociclib [10], and both in trials and in routine practice some patients progress within a few months whereas others remain on treatment for several years [2]. Host-related inflammatory indices such as the neutrophil-to-lymphocyte ratio have been associated with outcome in large real-world cohorts receiving CDK4/6 inhibitors [11], but no inexpensive and routinely available biomarker has been validated to identify, before treatment is initiated, those patients in whom the outcome is likely to be unfavourable, which represents a practical unmet need in an expanding treatment population.

Two host-related, blood-based indices are plausible candidates for this purpose. The Glasgow Prognostic Score (GPS) was first described by Forrest et al. in inoperable non-small-cell lung cancer and combines C-reactive protein and albumin, thereby reflecting the systemic inflammatory and nutritional state of the host [12]. In its original form, one point is assigned for each of an elevated C-reactive protein concentration (>10 mg/L) and hypoalbuminaemia (<35 g/L), so that patients fall into three categories (0, 1 and 2). In the original cohort of 161 patients, each one-point increase in the score was associated with a 1.7-fold increase in the hazard of death, a prognostic value comparable to that of the combination of stage and performance status [12]. In the modified version of the score, introduced subsequently, hypoalbuminaemia in the absence of an elevated C-reactive protein is not scored [13]. The score has since been validated across a wide range of tumour types and the available breast cancer evidence, derived largely from operable disease and from the modified version of the score, supports its prognostic value [14]. The LDH-to-albumin ratio (LAR) addresses the same question from a different perspective. Lactate dehydrogenase reflects the shift of proliferating tumour cells towards anaerobic glycolysis under hypoxic conditions, together with tumour burden and cell turnover, and elevated concentrations have repeatedly been associated with adverse outcomes in breast cancer [15,16,17]. Albumin, a negative acute-phase reactant, decreases in response both to malnutrition and to tumour-driven inflammation [18,19]. By relating a marker of tumour glycolytic activity to a marker of host inflammation and nutritional status, LAR combines these two domains within a single index derived from routine blood tests. Unlike GPS, LAR is a continuous ratio for which no standard risk categories have been established; in the studies that have evaluated it, thresholds were derived within each cohort, usually by receiver operating characteristic analysis, and they vary considerably between studies and with the units in which the two components are expressed. LAR has been associated with survival in colorectal, gastric and lung cancer [18,20,21,22,23]; a recent meta-analysis of 18 studies comprising more than 8000 patients confirmed its association with overall and progression-free survival across tumour types [24], and, in breast cancer, with pathological response following neoadjuvant chemotherapy, for which Savaş et al. identified an optimal threshold of 46.27 [25]. The two indices therefore share serum albumin, yet each combines it with a different component—C-reactive protein in the case of GPS and lactate dehydrogenase in the case of LAR—and there is no reason to assume that they convey equivalent prognostic information. The prognostic value of LAR in metastatic breast cancer treated with CDK4/6 inhibitors has nonetheless received little attention, and the two indices have not previously been evaluated within a single cohort. The present study therefore examined LAR and the original GPS in a consecutive series of patients with HR-positive, HER2-negative metastatic breast cancer treated with a CDK4/6 inhibitor, with overall survival as the primary and progression-free survival as the secondary endpoint.

## 2. Materials and Methods

### 2.1. Study Design and Participants

This single-centre retrospective cohort study included consecutive patients with hormone receptor-positive (HR+), HER2-negative metastatic breast cancer who began treatment with a CDK4/6 inhibitor (palbociclib or ribociclib) in combination with endocrine therapy, between January 2020 and April 2025. Reporting was guided by the STROBE statement for observational studies and by the REMARK recommendations for tumour marker prognostic studies.

Patients were eligible if they were aged 18 years or older, had histologically confirmed HR+ (oestrogen and/or progesterone receptor expression in ≥1% of tumour cells), HER2-negative metastatic breast cancer, had received at least one dose of a CDK4/6 inhibitor, and had baseline serum lactate dehydrogenase (LDH), albumin and C-reactive protein (CRP) measurements available. Patients with insufficient clinical or laboratory data, a second primary malignancy, an active infection or a chronic inflammatory disease were excluded, since each of these conditions independently alters LDH, albumin or CRP concentrations and would therefore confound both indices. The final cohort comprised 139 patients.

The study was approved by the Ethics Committee of Manisa Celal Bayar University (decision number E-20478486-050.04-1051689, dated 9 July 2025) and was conducted in accordance with the Declaration of Helsinki. Written informed consent was obtained from all patients or, for patients who had died, from their legal representatives, and patient data were anonymised before analysis.

### 2.2. Data Collection and Definitions

Demographic, clinicopathological, laboratory and treatment variables were extracted from electronic medical records and patient files. HER2 status was classified as HER2 IHC 0 or HER2-low (IHC 1+, or IHC 2+ with a negative ISH result); all patients were HER2-negative by conventional criteria. The Ki-67 proliferation index was analysed as a three-level categorical variable (1–20%, 20–50% and 50–99%) and, in the multivariable models, as a binary variable dichotomised at 50%. Visceral metastasis was defined as involvement of the liver, lung or brain. Endocrine sensitivity was classified according to the ESO–ESMO international consensus guidelines for advanced breast cancer [26]. Patients who relapsed during adjuvant endocrine therapy or within 12 months of its completion, and those who had received endocrine therapy for metastatic disease before the CDK4/6 inhibitor, were classified as endocrine-resistant. Patients with de novo metastatic disease who had not received prior endocrine therapy, and those who relapsed more than 12 months after completing adjuvant endocrine therapy or without having received it, were classified as endocrine-sensitive. As the start date of adjuvant endocrine therapy was not recorded, primary and secondary resistance were not distinguished. Metastatic burden was expressed as the number of involved sites among bone, lymph node, lung, liver and brain, and was dichotomised at three or more sites.

### 2.3. Index Prognostic Markers

Serum lactate dehydrogenase, albumin and C-reactive protein were determined from blood samples obtained immediately before the first dose of the CDK4/6 inhibitor; where more than one pretreatment measurement was available, the value closest to the initiation of therapy was used. Lactate dehydrogenase was expressed in U/L, albumin in g/dL and C-reactive protein in mg/L. Complete baseline values for all three parameters constituted an eligibility requirement, and no data were missing or imputed for any of the 139 patients.

LAR was analysed as a continuous variable in all survival models. Its distribution was markedly right-skewed (skewness 5.44), with two patients having values above 280, approximately six times the cohort median; a linear specification would therefore have been dominated by a small number of extreme observations and would have assigned equal multiplicative risk to absolute increments at the lower and upper ends of the distribution. LAR was accordingly modelled on the natural-logarithmic scale for both endpoints, with hazard ratios expressed per doubling and calculated as exp(β × ln 2). This specification was determined by the distribution of the marker and was independent of the outcome; results obtained on the untransformed scale are reported as a sensitivity analysis.

For the Kaplan–Meier analyses, patients were additionally dichotomised at a LAR of 46.27. No threshold for LAR has been established in metastatic breast cancer or in patients receiving CDK4/6 inhibitors, and the cut-offs reported in other malignancies vary with the population studied and with the units in which lactate dehydrogenase and albumin are expressed. The value of 46.27 was therefore not derived from the present dataset but was prespecified on the basis of a previously published breast cancer cohort in which the same units of measurement were used [25]. The use of an externally derived cut-off avoids the optimistic bias associated with selecting a threshold within the same data in which it is subsequently tested. The dichotomised variable was used for illustration only and did not replace continuous LAR in any Cox model. To examine whether the conclusions depended on this threshold, LAR was additionally analysed in tertiles of the present cohort, with a test for linear trend across tertiles, and the functional form of log-transformed LAR was assessed using a restricted cubic spline with three knots placed at the 10th, 50th and 90th percentiles.

The Glasgow Prognostic Score (GPS) was calculated in its original form [12]: patients with both an elevated CRP (>10 mg/L) and hypoalbuminaemia (<3.5 g/dL) were assigned a score of 2; those with only one of these abnormalities—including those with isolated hypoalbuminaemia—were assigned a score of 1; and those with neither were assigned a score of 0. The original score was chosen a priori in preference to the modified GPS, which assigns a score of 0 to isolated hypoalbuminaemia, so that albumin would contribute to both indices and the two could be compared on equivalent terms. As only six patients (4.3%) had a GPS of 2, the score was analysed primarily as a binary variable (GPS 0 versus GPS ≥ 1), the three-category form being presented as a supplementary analysis.

### 2.4. Endpoints and Follow-Up

The primary endpoint was overall survival (OS), measured from the first dose of the CDK4/6 inhibitor to death from any cause. The secondary endpoint was progression-free survival (PFS), measured from the first dose to radiologically or clinically documented disease progression or death from any cause, whichever occurred first. Progression was assessed by cross-sectional imaging according to the Response Evaluation Criteria in Solid Tumours (RECIST), version 1.1. Patients without an event were censored at the date on which they were last known to be alive and progression-free. Survival times were recorded in months, with sub-monthly durations expressed as fractions (for example, three weeks as 0.75 months).

The database was locked on 30 June 2026. By that date, 72 patients (51.8%) had died and 110 (79.1%) had experienced disease progression. The median duration of follow-up, estimated using the reverse Kaplan–Meier method, was 50.0 months (95% CI 44.1–56.0).

### 2.5. Statistical Analysis

Analyses were performed with IBM SPSS Statistics for Windows, version 27 (IBM Corp., Armonk, NY, USA), including the restricted cubic spline analysis, for which the spline term was computed as a derived variable and tested with a likelihood-ratio test; Harrell’s concordance index and the paired comparisons of concordance indices were performed in R, version 4.6.1, (R Foundation for Statistical Computing, Vienna, Austria). Categorical variables are presented as frequencies and percentages, and continuous variables as mean ± standard deviation or as median with interquartile range according to their distribution, which was assessed with the Shapiro–Wilk test. The association between the two indices was examined using Pearson’s chi-square test. All tests were two-sided and *p* < 0.05 was considered statistically significant. Given the exploratory nature of the analysis, no adjustment was made for multiple comparisons.

Survival was estimated by the Kaplan–Meier method and compared using the log-rank test. Variables were first examined in univariable Cox proportional-hazards models. As LAR and GPS both incorporate serum albumin and are therefore structurally collinear, the two indices were entered into separate multivariable models for each endpoint (Model A, LAR; Model B, GPS), each adjusted simultaneously for the same clinically prespecified covariates: lymph-node metastasis, visceral metastasis, a Ki-67 index ≥ 50% and the line of CDK4/6 inhibitor therapy. This yielded 14.4 events per variable for overall survival and 22.0 for progression-free survival. The proportional-hazards assumption was examined by log-minus-log plots for the binary covariates and by testing an interaction between each index and the natural logarithm of survival time. To assess residual confounding, both models were additionally adjusted, in a sensitivity analysis, for endocrine resistance, the presence of three or more metastatic sites and the endocrine partner (fulvestrant versus letrozole). These variables were not part of the prespecified primary models, and the extended models, with 9.0 events per variable for overall survival and 13.8 for progression-free survival, were regarded as exploratory.

The two indices were compared with respect to model fit, using the Akaike information criterion (lower values indicating better fit), and to discrimination, using Harrell’s concordance index with bootstrap confidence intervals together with a paired test of the difference between the two indices. Because a continuous variable is expected to outperform a categorical one in such comparisons by construction, the comparison was repeated on equal terms, first with both indices in binary form (LAR dichotomised at the prespecified threshold of 46.27 versus GPS 0 versus ≥ 1) and then with both in three-level form (tertiles of LAR versus the three original GPS categories).

## 3. Results

### 3.1. Patients and Baseline Characteristics

A total of 139 patients were analysed. The mean age was 53.73 ± 11.92 years (range 26–81); the cohort was almost exclusively female (*n* = 135, 97.1%) and predominantly postmenopausal (*n* = 99, 71.2%). Invasive ductal carcinoma was the dominant histology (*n* = 125, 89.9%), and most patients presented with systemic recurrence (*n* = 84, 60.4%) rather than de novo metastatic disease (*n* = 55, 39.6%). Bone was the most frequent metastatic site (*n* = 106, 76.3%), and visceral metastasis was present in 80 patients (57.6%). Eighty-four patients (60.4%) had HER2 IHC 0 and 55 (39.6%) HER2-low disease. Palbociclib and ribociclib were used in comparable proportions (51.1% and 48.9%), and the CDK4/6 inhibitor was administered as first-line therapy in 96 patients (69.1%) and as later-line therapy in 43 (30.9%). Letrozole was the endocrine partner in 83 patients (59.7%) and fulvestrant in 56 (40.3%). Ninety-one patients (65.5%) were classified as endocrine-resistant, including 79 of the 84 patients with systemic recurrence, and 48 (34.5%) as endocrine-sensitive; 45 patients (32.4%) had three or more metastatic sites. The mean LAR was 58.92 ± 35.83 (range 22.14–337.73); 82 patients (59.0%) fell above the prespecified threshold of 46.27 and 57 (41.0%) below it. The Glasgow Prognostic Score was 0 in 95 patients (68.3%), 1 in 38 (27.3%) and 2 in 6 (4.3%). The two indices were interrelated: a Glasgow Prognostic Score of 0 was recorded in 84.2% of patients with a LAR ≤ 46.27, compared with 57.3% of those above this threshold (*p* = 0.002), consistent with the albumin component that they share. Full baseline characteristics are presented in Table 1.

Over a median follow-up of 50.0 months (95% CI 44.1–56.0), 110 patients (79.1%) progressed and 72 (51.8%) died. Median overall survival for the whole cohort was 40.0 months (95% CI 30.0–50.0), and median progression-free survival was 19.0 months (95% CI 13.8–24.2). Median progression-free survival was 25.0 months (95% CI 20.1–29.9) in endocrine-sensitive and 16.0 months (95% CI 11.8–20.2) in endocrine-resistant patients (log-rank *p* = 0.475), and median overall survival was not reached and 35.0 months (95% CI 26.9–43.1), respectively (log-rank *p* = 0.036).

#### Kaplan–Meier Analyses

A high LAR was associated with shorter survival for both endpoints. Median overall survival was not reached in the low-LAR group, compared with 32.0 months (95% CI 24.9–39.1) in the high-LAR group (log-rank *p* = 0.001); estimated 1-, 2- and 3-year OS rates were 84.2%, 76.7% and 70.4% versus 81.7%, 65.1% and 44.6%, respectively (Figure 1A). Median progression-free survival was 28.0 months (95% CI 17.3–38.7) versus 16.0 months (95% CI 12.5–19.5) (log-rank *p* = 0.022), with 1-, 2- and 3-year PFS rates of 66.7%, 57.9% and 37.5% versus 59.8%, 30.9% and 15.7% (Figure 1C).

An elevated Glasgow Prognostic Score was associated with overall survival but not with progression-free survival. Median OS was 48.0 months (95% CI 35.6–60.4) for GPS 0 versus 30.0 months (95% CI 20.9–39.1) for GPS ≥ 1 (log-rank *p* = 0.027; Figure 1B), whereas median PFS was 19.0 months (95% CI 13.4–24.6) versus 14.0 months (95% CI 6.7–21.3) and did not differ significantly (log-rank *p* = 0.161; Figure 1D). Across the three original GPS categories, median OS declined stepwise (48.0, 30.0 and 3.0 months for GPS 0, 1 and 2; log-rank *p* = 0.025) and median PFS followed the same direction without reaching significance (19.0, 14.0 and 3.0 months; log-rank *p* = 0.125); the GPS 2 stratum comprised six patients, and the corresponding estimates are therefore unstable (Supplementary Figure S1).

### 3.2. Univariable Cox Regression

A higher LAR was associated with both endpoints: each doubling of LAR corresponded to an approximately threefold increase in the hazard of death (HR 3.048, 95% CI 1.894–4.908, *p* < 0.001) and a twofold increase in the hazard of progression (HR 2.140, 95% CI 1.413–3.242, *p* < 0.001). An elevated Glasgow Prognostic Score (≥1 versus 0) was associated with shorter overall survival (HR 1.710, 95% CI 1.054–2.775, *p* = 0.030) but not with progression-free survival (HR 1.320, 95% CI 0.888–1.963, *p* = 0.169).

Among the remaining variables, visceral, lymph-node and brain metastases and the presence of three or more metastatic sites were associated with adverse outcomes for both endpoints, whereas liver metastasis, a high Ki-67 index and later-line use of the CDK4/6 inhibitor were associated with shorter progression-free survival alone. Endocrine resistance was associated with shorter overall survival but not with progression-free survival. Age, sex, histopathology, mode of presentation, menopausal status, lung metastasis, progesterone-receptor status, HER2 status, the type of CDK4/6 inhibitor and the endocrine partner were not associated with either endpoint. The estimates for brain metastasis (*n* = 7) and male sex (*n* = 4) derive from small subgroups and have correspondingly wide confidence intervals. The full univariable results are presented in Table 2.

### 3.3. Multivariable Cox Regression

Because LAR and GPS both incorporate serum albumin, they were entered into two separate multivariable models for each endpoint (Model A: LAR; Model B: GPS), each adjusted for lymph-node metastasis, visceral metastasis, Ki-67 ≥ 50% and the line of CDK4/6 inhibitor therapy (Table 3).

The proportional-hazards assumption was satisfied in all four models: the time-dependent interaction between each index and the natural logarithm of survival time was non-significant throughout (LAR: *p* = 0.764 for OS and *p* = 0.403 for PFS; GPS: *p* = 0.279 and *p* = 0.417), and the log-minus-log plots for the binary covariates were approximately parallel.

LAR remained independently associated with both endpoints. In Model A, each doubling of LAR corresponded to a 2.87-fold increase in the hazard of death (adjusted HR 2.869, 95% CI 1.782–4.619, *p* < 0.001) and a 2.21-fold increase in the hazard of progression (adjusted HR 2.205, 95% CI 1.454–3.345, *p* < 0.001). GPS retained independent significance for overall survival alone (Model B: adjusted HR 1.719, 95% CI 1.047–2.820, *p* = 0.032) and remained non-significant for progression-free survival (adjusted HR 1.316, 95% CI 0.875–1.978, *p* = 0.187). In the progression-free survival models, lymph-node metastasis, visceral metastasis and later-line use of the CDK4/6 inhibitor also retained independent significance, whereas no covariate other than the index itself reached significance in the overall survival models.

In sensitivity analyses, the prespecified threshold of 46.27 lay close to the cohort median (49.75; 41st percentile). When LAR was categorised into tertiles of the present cohort (upper boundaries of the first and second tertiles, 44.6 and 57.2) and adjusted for the same covariates as Model A, the hazard increased stepwise from the lowest to the highest tertile for both overall survival (HR 1.80, 95% CI 0.96–3.39, and HR 2.41, 95% CI 1.28–4.51; *p* for trend = 0.006) and progression-free survival (HR 1.21, 95% CI 0.74–1.96, and HR 1.65, 95% CI 1.02–2.67; *p* for trend = 0.043). The restricted cubic spline showed no departure from log-linearity (*p* for non-linearity = 0.99 for overall survival and 0.25 for progression-free survival), indicating a graded association without a distinct threshold (Supplementary Table S1; Supplementary Figure S2).

In a further sensitivity analysis addressing residual confounding, both models were additionally adjusted for endocrine resistance, three or more metastatic sites and the endocrine partner (Supplementary Table S2). The association of LAR was essentially unchanged for both overall survival (adjusted HR 2.98 per doubling, 95% CI 1.80–4.93, *p* < 0.001) and progression-free survival (adjusted HR 2.09, 95% CI 1.36–3.22, *p* < 0.001). The association of GPS with overall survival, by contrast, was attenuated and no longer statistically significant (adjusted HR 1.37, 95% CI 0.82–2.31, *p* = 0.235). This attenuation was attributable mainly to metastatic burden: adjustment for three or more metastatic sites alone reduced the hazard ratio to 1.42 (95% CI 0.84–2.41, *p* = 0.191). In the extended models, endocrine resistance and three or more metastatic sites were independently associated with both endpoints.

The LAR-based models had lower AIC values than the GPS-based models for both endpoints (overall survival: model χ^2^ 29.0 vs. 16.7, AIC 612.3 vs. 624.6, ΔAIC 12.3; progression-free survival: model χ^2^ 35.6 vs. 24.7, AIC 906.4 vs. 917.3, ΔAIC 10.9).

### 3.4. Discrimination and Overall Comparison

Harrell’s concordance index was numerically higher for LAR than for GPS at both endpoints (overall survival: 0.609, 95% CI 0.538–0.686 versus 0.572, 95% CI 0.510–0.633; progression-free survival: 0.579, 95% CI 0.517–0.638 versus 0.540, 95% CI 0.489–0.590). The paired differences were small and did not reach statistical significance (ΔC ≈ 0.04; *p* > 0.20 for both endpoints), and the two indices therefore cannot be said to differ in discriminatory accuracy.

When the two indices were compared on equal terms, the differences between them diminished. With both indices in binary form, a LAR above 46.27 remained associated with both overall survival (adjusted HR 2.19, 95% CI 1.28–3.74, *p* = 0.004) and progression-free survival (adjusted HR 1.54, 95% CI 1.02–2.33, *p* = 0.039), but the AIC differences between the LAR- and GPS-based models fell to 4.5 and 2.7, and the concordance indices were almost identical (overall survival 0.581 vs. 0.572, *p* = 0.75; progression-free survival 0.554 vs. 0.540, *p* = 0.62). With both indices in three-level form, the AIC differences were 3.2 and 2.0, and the concordance indices were almost identical (overall survival 0.579 vs. 0.576, *p* = 0.96; progression-free survival 0.548 vs. 0.543, *p* = 0.91) (Supplementary Table S3).

Taken together, across Kaplan–Meier estimation and univariable and multivariable Cox regression, LAR was associated with both overall and progression-free survival, whereas GPS was associated with overall survival only; however, the two indices did not differ significantly in discrimination for either endpoint (Table 4).

## 4. Discussion

In this cohort of 139 patients with hormone receptor-positive, HER2-negative metastatic breast cancer treated with a CDK4/6 inhibitor, a higher baseline LDH-to-albumin ratio was independently associated with both shorter overall survival (adjusted HR 2.87 per doubling) and shorter progression-free survival (adjusted HR 2.21 per doubling), whereas an elevated Glasgow Prognostic Score was independently associated with overall survival alone (adjusted HR 1.72), an association that did not persist after additional adjustment for metastatic burden. Both indices therefore carried prognostic information in this setting, although they did so for different endpoints. LAR has not previously been examined in patients receiving CDK4/6 inhibitors, and the present analysis provides an initial estimate of its prognostic value in this population.

The strength of these associations should nevertheless be distinguished from the ability of either index to separate individual patients according to outcome. Discrimination was modest: the concordance index for LAR was 0.609 for overall survival and 0.579 for progression-free survival, only moderately above the value of 0.5 expected by chance and below the value of 0.70 often regarded as indicating acceptable discrimination. A hazard ratio of 2.87 per doubling therefore reflects a substantial difference in risk between groups of patients, but does not imply that LAR alone can reliably identify the individual patient in whom the outcome will be unfavourable. LAR is accordingly better regarded as one inexpensive component of a broader prognostic assessment, alongside established clinical factors such as the pattern of metastatic spread and the line of therapy, than as a stand-alone decision tool, and its incremental value over such factors would need to be established in larger cohorts. It should also be emphasised that LAR was evaluated here as a prognostic rather than a predictive biomarker. Because every patient in this cohort received a CDK4/6 inhibitor, the data show that a high LAR identifies patients with a less favourable outcome under this treatment, but cannot show whether such patients derive less benefit from the CDK4/6 inhibitor itself than they would from endocrine therapy alone. Establishing a predictive role would require comparison with patients treated without a CDK4/6 inhibitor, ideally through a test of the interaction between LAR and treatment within a randomised trial, and the present findings should not be used to select patients for, or withhold, CDK4/6 inhibitor therapy.

Two considerations may account for the divergent behaviour of the two indices with respect to progression. First, the components that they do not share address different biological domains: C-reactive protein reflects the systemic inflammatory response of the host, which is closely linked to cachexia, frailty and tolerance of therapy and therefore to mortality, whereas lactate dehydrogenase is more closely related to tumour burden and glycolytic activity, and hence to the tempo of disease progression under endocrine and CDK4/6 blockade. Second, the distribution of the Glasgow Prognostic Score in this relatively fit ambulatory population was strongly skewed towards its lowest category, 68.3% of patients scoring 0 and only 4.3% scoring 2. A three-level score in which two-thirds of patients occupy a single stratum has limited capacity to discriminate, particularly for an endpoint as frequent as progression. The difference observed between the two indices may therefore reflect measurement resolution as much as biology. Accordingly, the present data show that the two indices behaved differently with respect to progression, not that LAR is the superior prognostic marker. The lower AIC values of the LAR-based models were not accompanied by a significant difference in concordance, and a claim of superiority would require a demonstrable difference in discrimination, ideally in an independent cohort. This interpretation is supported by the comparisons made on equal terms: when both indices were entered in the same binary or three-level form, the difference in model fit largely disappeared and discrimination was similar, indicating that much of the apparent advantage of LAR in the primary analysis derived from its continuous scale. The association of LAR with progression-free survival, however, persisted when it was dichotomised.

The prognostic value of LAR observed here is consistent with reports in other tumour types. In colorectal cancer, a high preoperative LAR has been associated with poor survival in two independent series [18,20], and in colon cancer Xie et al. reported three-year survival of 56.0% among patients with a high LAR compared with 90.9% among those with a low LAR [22]. Comparable findings have been described in patients receiving immune checkpoint inhibitors: LAR was associated with adverse outcomes in gastric cancer treated with nivolumab [23], and in non-small-cell lung cancer. Menekşe et al. identified LAR as the only independent prognostic factor for progression-free survival and, together with performance status, for overall survival [21]. Evidence in breast cancer remains limited and derives predominantly from non-metastatic disease. Savaş et al. examined LAR in patients receiving neoadjuvant chemotherapy and identified 46.27 as the optimal threshold for predicting pathological complete response [25], the value adopted as the external cut-off in the present analysis, and Tan et al. reported that the GRIm score, which likewise combines lactate dehydrogenase and albumin, was associated with both overall and progression-free survival in early breast cancer [27]. The individual components have been investigated more extensively: elevated LDH has been linked to adverse outcomes in metastatic breast cancer in several cohorts and in a meta-analysis [15,16,17], and hypoalbuminaemia has shown comparable associations in non-metastatic disease [19]. Composite indices derived from routine laboratory parameters have attracted comparable interest in this disease: Eniseler et al. reported that a score combining CA 15-3 with the neutrophil-to-lymphocyte ratio predicted pathological complete response following neoadjuvant chemotherapy [28], reflecting a broader effort to extract prognostic information from investigations already performed in routine practice. The present findings extend this body of work to the metastatic, CDK4/6-inhibitor-treated setting.

The overall survival finding for the Glasgow Prognostic Score accords with the established literature. Since its original description in inoperable non-small-cell lung cancer [12], the score has been validated across a wide range of malignancies, and the available breast cancer evidence, derived largely from operable disease and from the modified version of the score, supports its association with overall survival [14]. Its relationship to progression has been examined less frequently, as most studies of inflammation-based scores in breast cancer have adopted survival or recurrence as the sole endpoint. The original rather than the modified score was used here so that albumin would contribute to both indices in the same manner; under the modified score, patients with isolated hypoalbuminaemia are assigned a score of 0, which would have removed from GPS the component that it shares with LAR.

The sensitivity analysis addressing residual confounding qualifies this finding. When the models were additionally adjusted for endocrine resistance, metastatic burden and the endocrine partner, the association of LAR with both endpoints was essentially unchanged, whereas that of GPS with overall survival was attenuated and lost statistical significance, mainly as a result of adjustment for the number of metastatic sites. This suggests that part of the prognostic information conveyed by GPS in this cohort reflected the extent of metastatic disease, which is itself associated with systemic inflammation, whereas the information conveyed by LAR was not explained by the clinical variables available. The extended models were exploratory, with fewer events per variable than the primary models, and this contrast does not in itself establish that LAR is the better marker.

Endocrine sensitivity also provides context for the survival observed in this cohort. The median progression-free survival of 19.0 months lies between the values reported in the pivotal trials: in PALOMA-2, which enrolled endocrine-sensitive patients receiving first-line palbociclib and letrozole, median progression-free survival was 24.8 months [7], whereas in PALOMA-3, which enrolled patients whose disease had progressed on prior endocrine therapy, it was 9.5 months with palbociclib and fulvestrant [29]. Two-thirds of the present cohort were endocrine-resistant, and median progression-free survival was 25.0 months among endocrine-sensitive patients, closely matching the first-line trial population, compared with 16.0 months among endocrine-resistant patients. The difference between these subgroups did not reach statistical significance in univariable analysis, but endocrine resistance was independently associated with shorter progression-free survival once metastatic burden and the other covariates had been taken into account. The overall figure of 19.0 months therefore reflects the mixed composition of a real-world cohort rather than an unusually favourable outcome.

Two secondary observations merit brief mention. The specific CDK4/6 inhibitor received was not associated with either endpoint (ribociclib versus palbociclib: OS HR 0.79, *p* = 0.339; PFS HR 0.76, *p* = 0.153). The study was neither designed nor powered for this comparison and treatment was not randomly allocated, so this result should not be interpreted as evidence of equivalence. HER2-low status was likewise not associated with either endpoint, in keeping with evidence that it does not constitute an independent prognostic category in hormone receptor-positive disease.

Certain methodological strengths merit note. The LAR threshold was prespecified from an external cohort rather than derived from the present data, and the dichotomised variable was used for illustration only, with all inferential models treating LAR as a continuous variable. Both indices were evaluated in the same cohort, with the same covariates and the same endpoints, so that the comparison is not confounded by differences in case mix or model specification. Follow-up was mature, and the proportional-hazards assumption was verified in every model.

## 5. Limitations

This study has several limitations. First, it is a retrospective analysis of 139 patients treated at a single university hospital, and the findings may not be generalisable to populations with different patient characteristics, treatment practices or laboratory methods. The modest sample size also limited the precision of the estimates, as reflected in the width of several confidence intervals, and did not permit the cohort to be divided into derivation and validation subsets. The findings therefore require external validation in larger, preferably multicentre cohorts before they can inform clinical decisions. As screening and exclusion counts could not be reconstructed from the records, selection bias cannot be excluded. Lactate dehydrogenase, albumin and C-reactive protein are all influenced by conditions other than malignancy, and although patients with active infection, chronic inflammatory disease or a second primary malignancy were excluded, residual confounding remains possible. Performance status and comorbidities were not consistently recorded in the retrospective records and could not be included in the models; both are associated with survival and with the laboratory components of the two indices, and their omission may have introduced residual confounding. In addition, the start date of adjuvant endocrine therapy was unavailable, so that primary and secondary endocrine resistance could not be distinguished. In addition, the start date of adjuvant endocrine therapy was unavailable, so that primary and secondary endocrine resistance could not be distinguished. The analysis was also restricted to baseline values; whether changes in LAR during treatment carry additional prognostic information, as has been reported for lactate dehydrogenase in metastatic breast cancer [16] and, more recently, for the neutrophil-to-lymphocyte ratio during CDK4/6 inhibitor therapy [30], merits investigation. The analyses were exploratory and no adjustment was made for multiple comparisons.

A structural asymmetry between the two indices should also be noted. LAR is a continuous variable, whereas GPS is a three-level ordinal score, and comparisons of model fit and discrimination in the primary analysis therefore favour LAR by construction. The comparisons made on equal terms indicate that this asymmetry accounts for much of the difference in model fit between the two indices, and the primary comparison should be interpreted in this light. Only six patients had a Glasgow Prognostic Score of 2, so the three-category estimates are unstable, and the binary form of the score was accordingly prespecified as the primary analysis. The distribution of LAR was dominated by two extreme observations and the marker was modelled on the logarithmic scale; results on the untransformed scale were concordant. Finally, the threshold of 46.27 was derived in a neoadjuvant, non-metastatic population, and its transportability to the metastatic setting remains an assumption. This assumption affects the Kaplan–Meier analyses alone, since LAR entered every regression model as a continuous variable, and the graded association observed across tertiles of the present cohort indicates that the findings did not depend on the choice of threshold. A threshold suitable for clinical use would need to be defined and validated in independent metastatic cohorts.

## 6. Conclusions

In patients with hormone receptor-positive, HER2-negative metastatic breast cancer receiving CDK4/6 inhibitors, the baseline LDH-to-albumin ratio, an inexpensive index derived from routine blood tests, was independently associated with both overall and progression-free survival, and this association persisted after additional adjustment for endocrine resistance, metastatic burden and the endocrine partner. The Glasgow Prognostic Score was associated with overall survival alone, an association that was attenuated after adjustment for metastatic burden. The discriminatory accuracy of both indices was modest; the two could not be shown to differ, and the present data do not establish the superiority of either. LAR should therefore be regarded as a candidate prognostic marker rather than a tool for clinical decision-making, and it provides no information on the benefit derived from CDK4/6 inhibition itself. Its value needs to be confirmed in independent, preferably multicentre, cohorts, and we would encourage other investigators to examine it in their own series using the continuous specification and the prespecified threshold reported here. Such studies could also address whether changes in LAR during treatment carry information beyond the baseline value, for example as an early marker of response or progression that could complement radiological assessment.

## Figures and Tables

**Figure 1:**
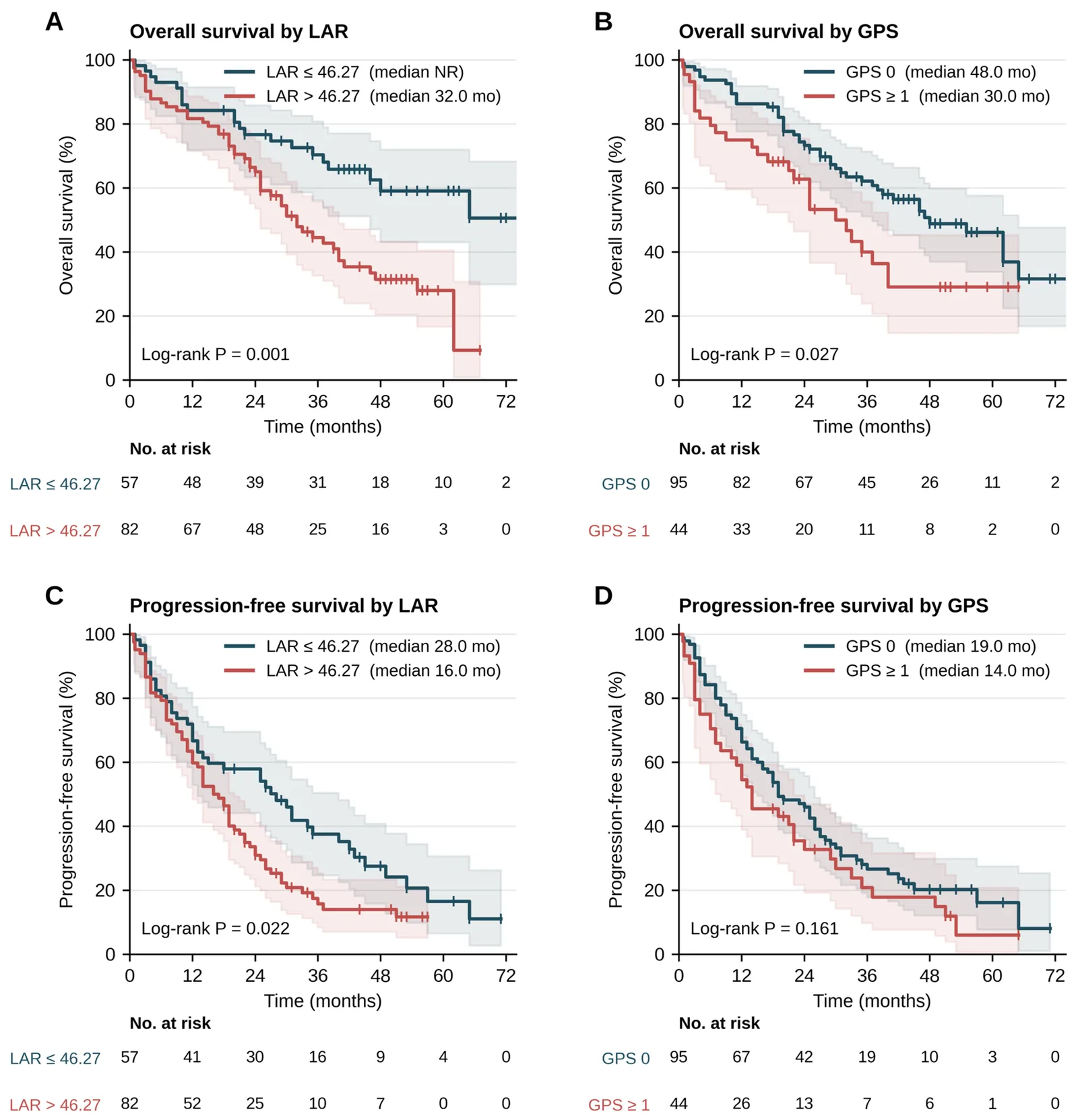
Kaplan–Meier curves for overall survival (**A**,**B**) and progression-free survival (**C**,**D**) according to the LDH-to-albumin ratio (LAR; **A**,**C**) and the Glasgow Prognostic Score (GPS; **B**,**D**) in 139 patients with HR-positive, HER2-negative metastatic breast cancer treated with a CDK4/6 inhibitor. Shaded areas indicate 95% confidence intervals and tick marks indicate censored observations; *p*-values were derived from the log-rank test. Median survival times are given in months (mo). NR, not reached.

**Table 1:** Baseline Characteristics, Treatment and Outcomes of the Study Cohort (*n* = 139).

Characteristic	Total Cohort (*n* = 139)
***Continuous variables, mean ± SD***	
**Age, years**	**53.73 ± 11.92**
**LDH-to-albumin ratio**	**58.92 ± 35.83**
***Demographic characteristics, n (%)***	
**Sex**	
*Female*	135 (97.1)
*Male*	4 (2.9)
**Menopausal status**	
*Premenopausal*	36 (25.9)
*Postmenopausal*	99 (71.2)
*Not applicable (male)*	4 (2.9)
***Tumour characteristics, n (%)***	
**Histopathology**	
*Invasive ductal carcinoma*	125 (89.9)
*Other*	14 (10.1)
**Mode of presentation**	
*De novo metastatic*	55 (39.6)
*Systemic recurrence*	84 (60.4)
**Oestrogen receptor**	
*Negative*	1 (0.7)
*1–10%*	4 (2.9)
*10–50%*	11 (7.9)
*50–90%*	61 (43.9)
*>90%*	62 (44.6)
**Progesterone receptor**	
*Negative*	49 (35.3)
*1–90%*	71 (51.1)
*>90%*	19 (13.7)
**Ki-67 index**	
*1–20%*	60 (43.2)
*20–50%*	56 (40.3)
*50–99%*	23 (16.5)
**HER2 immunohistochemistry ^a^**	
*IHC 0*	84 (60.4)
*IHC 1+*	32 (23.0)
*IHC 2+/ISH negative*	23 (16.5)
***Metastatic sites (present), n (%)***	
*Bone*	106 (76.3)
*Lymph node*	79 (56.8)
*Lung*	54 (38.8)
*Liver*	32 (23.0)
*Brain*	7 (5.0)
*Visceral ^b^*	80 (57.6)
*≥3 sites ^c^*	45 (32.4)
***Treatment characteristics, n (%)***	
**Line of CDK4/6 inhibitor therapy**	
*First-line*	96 (69.1)
*Later-line*	43 (30.9)
**CDK4/6 inhibitor**	
*Palbociclib*	71 (51.1)
*Ribociclib*	68 (48.9)
**Endocrine partner**	
*Letrozole*	83 (59.7)
*Fulvestrant*	56 (40.3)
**Endocrine sensitivity ^d^**	
*Endocrine-sensitive*	48 (34.5)
*Endocrine-resistant*	91 (65.5)
***Index prognostic markers, n (%)***	
**LAR group ^e^**	
*≤46.27*	57 (41.0)
*>46.27*	82 (59.0)
**Glasgow Prognostic Score**	
*0*	95 (68.3)
*1*	38 (27.3)
*2*	6 (4.3)
***Outcomes***	
**Median follow-up, months (95% CI) ^f^**	**50.0 (44.1–56.0)**
**Median progression-free survival, months (95% CI)**	**19.0 (13.8–24.2)**
**Median overall survival, months (95% CI)**	**40.0 (30.0–50.0)**
**Disease progression, *n* (%)**	**110 (79.1)**
**Death, *n* (%)**	**72 (51.8)**

^a^ All patients were HER2-negative by conventional criteria; those with IHC 1+ or IHC 2+/ISH-negative results are classified as HER2-low and those with IHC 0 are reported separately. ^b^ Visceral metastasis was defined as involvement of the liver, lung or brain. ^c^ Number of involved sites among bone, lymph node, lung, liver and brain. ^d^ Classified according to the ESO–ESMO ABC criteria (see Section 2). ^e^ LAR was dichotomised at the prespecified threshold of 46.27 for descriptive purposes and for the Kaplan–Meier analyses; it was modelled as a continuous variable in all Cox models. ^f^ Estimated by the reverse Kaplan–Meier method. Abbreviations: CDK, cyclin-dependent kinase; CI, confidence interval; IHC, immunohistochemistry; ISH, in situ hybridisation; LAR, lactate dehydrogenase-to-albumin ratio; LDH, lactate dehydrogenase; SD, standard deviation.

**Table 2:** Univariable Cox Regression Analyses for Overall and Progression-Free Survival (*n* = 139).

Variable	Overall Survival (72 Events)		Progression-Free Survival (110 Events)	
	HR (95% CI)	*p*	HR (95% CI)	*p*
***Prognostic markers***				
**LAR, per doubling ^a^**	**3.048 (1.894–4.908)**	**<0.001**	**2.140 (1.413–3.242)**	**<0.001**
**GPS ≥ 1 vs. 0**	**1.710 (1.054–2.775)**	**0.030**	**1.320 (0.888–1.963)**	**0.169**
***Metastatic sites***				
*Visceral*	1.863 (1.134–3.058)	**0.014**	1.789 (1.210–2.645)	**0.004**
*Lymph node*	1.829 (1.113–3.004)	**0.017**	1.914 (1.286–2.848)	**0.001**
*Bone*	1.743 (0.982–3.092)	0.058	1.488 (0.943–2.348)	0.087
*Liver*	1.545 (0.933–2.557)	0.091	1.634 (1.067–2.502)	**0.024**
*Lung*	1.282 (0.800–2.054)	0.302	1.239 (0.845–1.816)	0.272
*Brain ^b^*	3.845 (1.646–8.979)	**0.002**	3.214 (1.464–7.056)	**0.004**
*≥3 metastatic sites*	2.750 (1.704–4.438)	**<0.001**	2.648 (1.787–3.923)	**<0.001**
***Tumour and treatment characteristics***				
**Ki-67 index, overall**	**—**	**0.450**	**—**	**0.031**
*20–50% vs. 1–20%*	1.227 (0.734–2.051)	0.435	1.391 (0.919–2.106)	0.119
*50–99% vs. 1–20%*	1.519 (0.781–2.954)	0.218	2.045 (1.188–3.519)	**0.010**
*≥50% vs. <50% ^c^*	1.366 (0.747–2.498)	0.312	1.735 (1.058–2.844)	**0.029**
*CDK4/6 inhibitor line (later-line vs. first-line)*	1.430 (0.886–2.309)	0.143	1.570 (1.065–2.313)	**0.023**
*CDK4/6 inhibitor (ribociclib vs. palbociclib)*	0.794 (0.495–1.274)	0.339	0.759 (0.519–1.109)	0.153
*Endocrine partner (fulvestrant vs. letrozole)*	1.137 (0.711–1.818)	0.592	1.102 (0.751–1.617)	0.619
*Endocrine resistance (resistant vs. sensitive)*	1.791 (1.025–3.128)	**0.041**	1.154 (0.774–1.719)	0.482
*HER2 status (IHC 0 vs. HER2-low)*	1.016 (0.633–1.629)	0.949	1.279 (0.863–1.895)	0.219
*Progesterone receptor (negative vs. positive)*	0.816 (0.497–1.340)	0.422	1.154 (0.782–1.702)	0.471
*Histopathology (IDC vs. other)*	1.071 (0.463–2.476)	0.872	1.223 (0.618–2.422)	0.563
***Demographic characteristics***				
*Age, per year*	1.002 (0.983–1.022)	0.827	0.998 (0.983–1.013)	0.778
*Sex (male vs. female) ^b^*	2.591 (0.941–7.130)	0.065	2.099 (0.767–5.743)	0.149
*Menopausal status (post- vs. premenopausal)*	1.334 (0.760–2.341)	0.316	1.267 (0.813–1.973)	0.296
*Presentation (recurrence vs. de novo)*	1.407 (0.851–2.326)	0.184	1.179 (0.803–1.731)	0.402

^a^ LAR was markedly right-skewed (skewness 5.44) and was therefore modelled on the natural-logarithmic scale; the hazard ratio is expressed per doubling of LAR, calculated as exp(β × ln 2). On the untransformed linear scale, the corresponding hazard ratios per 10-unit increase were 1.176 (1.109–1.246) for overall survival and 1.157 (1.094–1.225) for progression-free survival, both *p* < 0.001; the conclusions are therefore unaffected by the choice of scale. ^b^ Estimates are based on small subgroups (brain metastasis, *n* = 7; male sex, *n* = 4) and have wide confidence intervals. ^c^ Ki-67 was additionally analysed as a binary variable (≥50% vs. <50%), which was the form entered into the multivariable models (Table 3). Bold *p*-values denote statistical significance (*p* < 0.05). Abbreviations: CDK, cyclin-dependent kinase; CI, confidence interval; GPS, Glasgow Prognostic Score; HR, hazard ratio; IDC, invasive ductal carcinoma; LAR, lactate dehydrogenase-to-albumin ratio.

**Table 3:** Multivariable Cox Regression Analyses for Overall and Progression-Free Survival (*n* = 139).

Variable	Model A (LAR)		Model B (GPS)	
	Adjusted HR (95% CI)	*p*	Adjusted HR (95% CI)	*p*
***Overall survival (n = 139, 72 events)***				
**LAR, per doubling ^a^**	**2.869 (1.782–4.619)**	**<0.001**	**—**	**—**
**GPS ≥ 1 vs. 0**	**—**	**—**	**1.719 (1.047–2.820)**	**0.032**
*Lymph-node metastasis*	1.462 (0.868–2.465)	0.153	1.523 (0.902–2.571)	0.115
*Visceral metastasis*	1.552 (0.928–2.595)	0.094	1.642 (0.981–2.749)	0.059
*Ki-67 ≥ 50%*	1.077 (0.578–2.007)	0.814	1.186 (0.646–2.178)	0.583
*Later-line CDK4/6 inhibitor*	1.594 (0.980–2.591)	0.060	1.509 (0.931–2.448)	0.095
**Model χ^2^ (df = 5)**	**29.0**	**<0.001**	**16.7**	**0.005**
**AIC**	**612.3**	**—**	**624.6**	**—**
***Progression-free survival (n = 139, 110 events)***				
**LAR, per doubling ^a^**	**2.205 (1.454–3.345)**	**<0.001**	**—**	**—**
**GPS ≥ 1 vs. 0**	**—**	**—**	**1.316 (0.875–1.978)**	**0.187**
*Lymph-node metastasis*	1.603 (1.055–2.435)	**0.027**	1.647 (1.079–2.516)	**0.021**
*Visceral metastasis*	1.505 (1.002–2.261)	**0.049**	1.542 (1.024–2.321)	**0.038**
*Ki-67 ≥ 50%*	1.441 (0.870–2.386)	0.156	1.437 (0.870–2.371)	0.156
*Later-line CDK4/6 inhibitor*	1.793 (1.196–2.689)	**0.005**	1.607 (1.080–2.390)	**0.019**
**Model χ^2^ (df = 5)**	**35.6**	**<0.001**	**24.7**	**<0.001**
**AIC**	**906.4**	**—**	**917.3**	**—**

Because LAR and GPS both incorporate serum albumin, they were entered into two separate multivariable Cox models (Model A and Model B), each adjusted for the same prespecified covariates (lymph-node metastasis, visceral metastasis, Ki-67 ≥ 50%, and the line of CDK4/6 inhibitor therapy; Enter method)—indicates that the marker was not included in the corresponding model. ^a^ LAR was modelled on the natural-logarithmic scale, with the hazard ratio expressed per doubling of LAR. The proportional-hazards assumption was satisfied in all four models (time-dependent interaction with the natural logarithm of survival time, *p* = 0.28–0.76). AIC = −2 log-likelihood + 2k, where k is the number of covariates; a lower AIC indicates better model fit (ΔAIC = 12.3 for overall survival and 10.9 for progression-free survival, both lower for the LAR-based model). Bold *p*-values denote statistical significance (*p* < 0.05). Abbreviations: AIC, Akaike information criterion; CDK, cyclin-dependent kinase; CI, confidence interval; GPS, Glasgow Prognostic Score; HR, hazard ratio; LAR, lactate dehydrogenase-to-albumin ratio.

**Table 4:** Prognostic Performance of LAR and GPS for Overall and Progression-Free Survival (*n* = 139).

Analysis	Overall Survival		Progression-Free Survival	
	LAR	GPS	LAR	GPS
***Kaplan–Meier analysis***				
*Median survival (months) ^a^*	NR vs. 32.0	48.0 vs. 30.0	28.0 vs. 16.0	19.0 vs. 14.0
*Log-rank p*	**0.001**	**0.027**	**0.022**	0.161
***Univariable Cox regression***				
*HR (95% CI)*	3.048 (1.894–4.908)	1.710 (1.054–2.775)	2.140 (1.413–3.242)	1.320 (0.888–1.963)
*p*	**<0.001**	**0.030**	**<0.001**	0.169
***Multivariable Cox regression***				
*Adjusted HR (95% CI)*	2.869 (1.782–4.619)	1.719 (1.047–2.820)	2.205 (1.454–3.345)	1.316 (0.875–1.978)
*p*	**<0.001**	**0.032**	**<0.001**	0.187
***Model fit***				
*Model χ^2^ (df = 5)*	29.0	16.7	35.6	24.7
*AIC*	612.3	624.6	906.4	917.3
*ΔAIC (GPS − LAR)*	**12.3**		**10.9**	
***Discrimination***				
*C-index (95% CI)*	0.609 (0.538–0.686)	0.572 (0.510–0.633)	0.579 (0.517–0.638)	0.540 (0.489–0.590)
*ΔC-index (LAR − GPS)*	0.037		0.039	
*p (paired comparison)*	NS (*p* > 0.20)		NS (*p* > 0.20)	

^a^ Median survival is shown as the low-risk versus the high-risk group (LAR ≤ 46.27 vs. >46.27; GPS 0 vs. ≥1). LAR was modelled on the natural-logarithmic scale throughout, and all LAR hazard ratios are expressed per doubling of LAR. Multivariable models were adjusted for lymph-node metastasis, visceral metastasis, Ki-67 ≥ 50%, and the line of CDK4/6 inhibitor therapy. A lower AIC indicates better model fit. C-index denotes Harrell’s concordance index with bootstrap confidence intervals; the paired between-marker difference was not statistically significant for either endpoint (*p* > 0.20). Bold *p*-values denote statistical significance (*p* < 0.05). Abbreviations: AIC, Akaike information criterion; CI, confidence interval; GPS, Glasgow Prognostic Score; HR, hazard ratio; LAR, lactate dehydrogenase-to-albumin ratio; NR, not reached; NS, not significant.

## Data Availability

The dataset generated and analyzed during the current study is available from the corresponding author upon reasonable request.

## Supplementary Materials

The following supporting information can be downloaded at: https://www.mdpi.com/article/10.3390/curroncol33100582/s1, Figure S1: Kaplan–Meier curves for (A) overall survival and (B) progression-free survival according to the three original categories of the Glasgow Prognostic Score (GPS 0, *n* = 95; GPS 1, *n* = 38; GPS 2, *n* = 6) in 139 patients with HR-positive, HER2-negative metastatic breast cancer treated with a CDK4/6 inhibitor. Shaded areas indicate 95% confidence intervals and tick marks indicate censored observations; *p* values were derived from the log-rank test. Median survival times are given in months (mo). Estimates for GPS 2 are based on six patients and are therefore unstable; Figure S2: Adjusted association between the LDH-to-albumin ratio and (A) overall survival and (B) progression-free survival, modelled with a restricted cubic spline of log-transformed LAR (knots at the 10th, 50th and 90th percentiles). Hazard ratios are relative to the cohort median (49.75) and adjusted for lymph-node metastasis, visceral metastasis, Ki-67 ≥ 50% and the line of CDK4/6 inhibitor therapy; shaded areas denote 95% confidence intervals. The dotted line marks the external threshold of 46.27, and tick marks along the x-axis show individual observations. The x-axis is truncated at 150 for legibility (two observations lie beyond this value); Table S1: Sensitivity analyses of the association between the LDH-to-albumin ratio and survival (*n* = 139); Table S2: Multivariable Cox regression models additionally adjusted for endocrine resistance, metastatic burden and endocrine partner (*n* = 139); Table S3: Comparison of LAR and GPS on equal terms (*n* = 139).
