# Supplementary material for: Lactate Dehydrogenase-to-Albumin Ratio and Glasgow Prognostic Score as Prognostic Markers in Metastatic Breast Cancer Treated with CDK4/6 Inhibitors
Source: Curr Oncol. 2026 Sep 29;33(10):582. doi: 10.3390/curroncol33100582 (PMC13703568; doi:10.3390/curroncol33100582)
Supplement: Supplementary file 1 [file curroncol-33-00582-s001.zip › curroncol-4572037-supplementary.pdf]

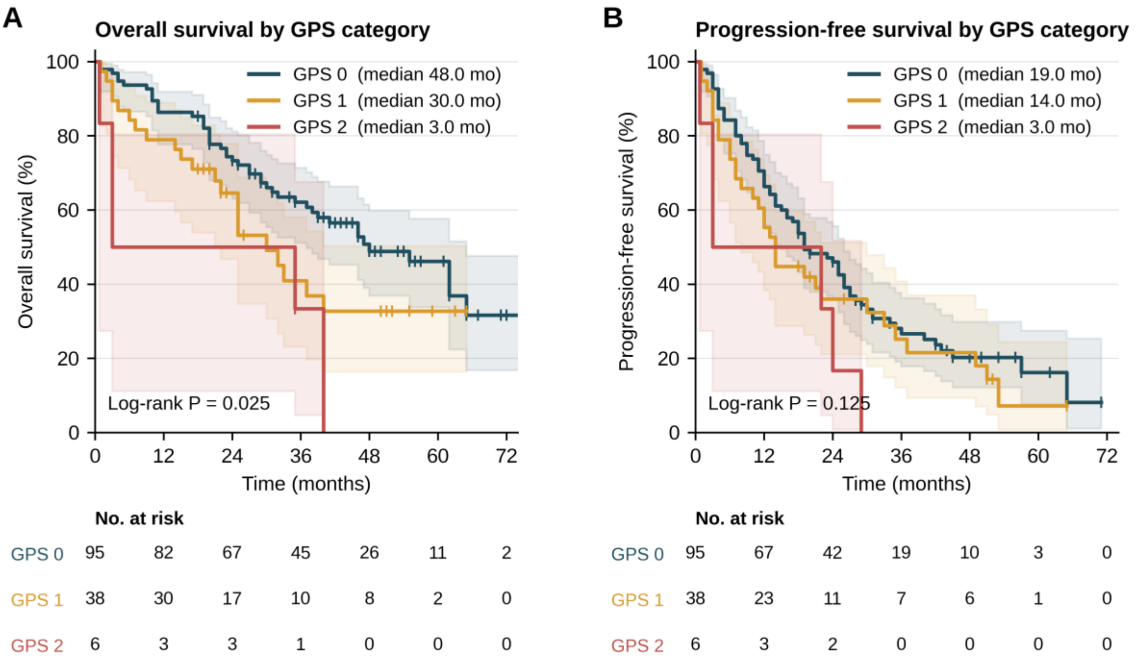

Figure S1. Kaplan–Meier curves for (A) overall survival and (B) progression-free survival according to the three original categories of the Glasgow Prognostic Score (GPS 0, n = 95; GPS 1, n = 38; GPS 2, n = 6) in 139 patients with HR-positive, HER2-negative metastatic breast cancer treated with a CDK4/6 inhibitor. Shaded areas indicate 95% confidence intervals and tick marks indicate censored observations; p values were derived from the log-rank test. Median survival times are given in months (mo). Estimates for GPS 2 are based on six patients and are therefore unstable.

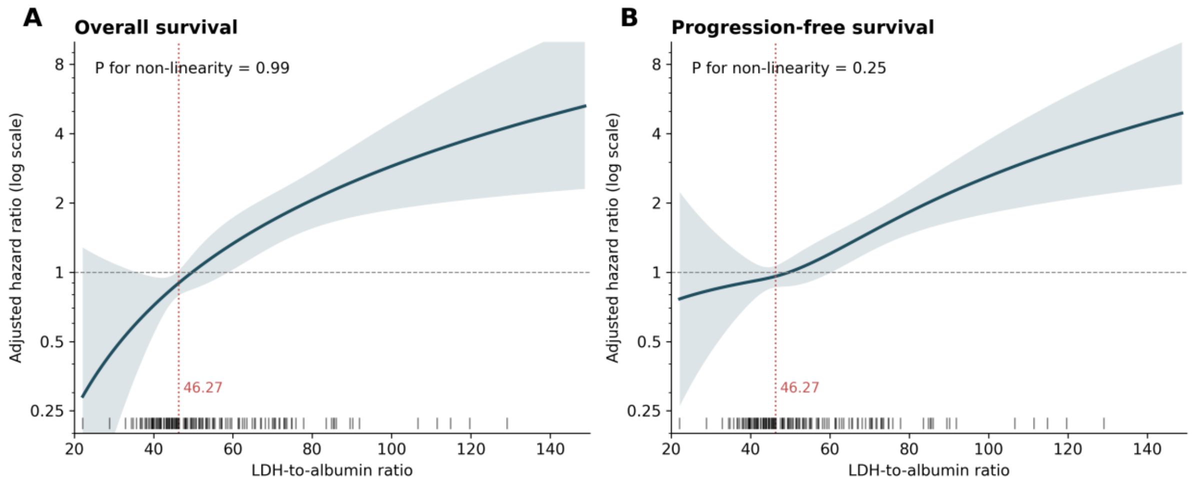

Figure S2. Adjusted association between the LDH-to-albumin ratio and (A) overall survival and (B) progression-free survival, modelled with a restricted cubic spline of log-transformed LAR (knots at the 10th, 50th and 90th percentiles). Hazard ratios are relative to the cohort median (49.75) and adjusted for lymph-node metastasis, visceral metastasis, Ki-67 ≥50% and the line of CDK4/6 inhibitor therapy; shaded areas denote 95% confidence intervals. The dotted line marks the external threshold of 46.27, and tick marks along the x-axis show individual observations. The x-axis is truncated at 150 for legibility (two observations lie beyond this value).

Table S1. Sensitivity Analyses of the Association Between the LDH-to-Albumin Ratio and Survival (n = 139).

LAR	Overall survival (72 events)		Progression-free survival (110 events)	
	**HR (95% CI)**	**p**	**HR (95% CI)**	**p**
	***LAR tertiles*[a]**			
Tertile 1 (22.14–44.62), n = 46	1.000 (reference)	—	1.000 (reference)	—
Tertile 2 (44.65–57.21), n = 47	1.800 (0.957–3.385)	0.068	1.208 (0.744–1.961)	0.446
Tertile 3 (58.16–337.73), n = 46	2.407 (1.284–4.513)	**0.006**	1.647 (1.015–2.670)	**0.043**
p for trend across tertiles[b]	—	**0.006**	—	**0.043**
	Continuous LAR			
LAR, per doubling (Model A)[c]	2.869 (1.782–4.619)	**<0.001**	2.205 (1.454–3.345)	**<0.001**
Non-linearity (restricted cubic spline)[d]	—	0.989	—	0.250

All models were adjusted for lymph-node metastasis, visceral metastasis, Ki-67 ≥50% and the line of CDK4/6 inhibitor therapy (Enter method), as in Model A (Table 3). [a] Tertiles were defined within the present cohort; the numbers of deaths and progression events were 16 and 36 in the first, 26 and 34 in the second, and 30 and 40 in the third tertile. [b] Tertile entered as an ordinal variable (1–3). [c] LAR was modelled on the natural-logarithmic scale, with the hazard ratio expressed per doubling of LAR; shown for comparison with Table 3. [d] Likelihood-ratio test comparing a restricted cubic spline of log-transformed LAR (knots at the 10th, 50th and 90th percentiles) with the log-linear specification. Bold p-values denote statistical significance ($p < 0.05$). Abbreviations: CDK, cyclin-dependent kinase; CI, confidence interval; HR, hazard ratio; LAR, lactate dehydrogenase-to-albumin ratio; LDH, lactate dehydrogenase.

Table S2. Multivariable Cox Regression Models Additionally Adjusted for Endocrine Resistance, Metastatic Burden and Endocrine Partner (n = 139).

Variable	Extended Model A (LAR)		Extended Model B (GPS)	
	Adjusted HR (95% CI)	p	Adjusted HR (95% CI)	p
Overall survival (n = 139, 72 events)				
**LAR, per doubling[a]**	**2.976 (1.795–4.934)**	**<0.001**	—	—
**GPS ≥1 vs 0**	—	—	**1.371 (0.815–2.305)**	**0.235**
Lymph-node metastasis	1.270 (0.655–2.463)	0.479	1.334 (0.686–2.595)	0.396
Visceral metastasis	0.830 (0.438–1.570)	0.566	0.962 (0.507–1.827)	0.906
Ki-67 ≥50%	1.593 (0.835–3.041)	0.158	1.468 (0.782–2.756)	0.232
Later-line CDK4/6 inhibitor	1.210 (0.740–1.979)	0.446	1.204 (0.739–1.963)	0.456
Endocrine resistance[b]	4.266 (2.047–8.888)	**<0.001**	3.648 (1.788–7.444)	**<0.001**
≥3 metastatic sites[c]	3.332 (1.545–7.186)	**0.002**	3.194 (1.450–7.036)	**0.004**
Fulvestrant vs letrozole	0.546 (0.312–0.955)	**0.034**	0.702 (0.402–1.228)	0.215
Progression-free survival (n = 139, 110 events)				
**LAR, per doubling[a]**	**2.092 (1.358–3.223)**	**<0.001**	—	—
**GPS ≥1 vs 0**	—	—	**1.028 (0.664–1.591)**	**0.902**
Lymph-node metastasis	1.319 (0.796–2.186)	0.283	1.382 (0.833–2.293)	0.210
Visceral metastasis	1.017 (0.614–1.684)	0.948	1.019 (0.609–1.704)	0.944
Ki-67 ≥50%	1.608 (0.949–2.722)	0.077	1.496 (0.889–2.517)	0.129
Later-line CDK4/6 inhibitor	1.538 (1.016–2.328)	**0.042**	1.395 (0.922–2.108)	0.115
Endocrine resistance[b]	1.977 (1.109–3.527)	**0.021**	1.985 (1.109–3.552)	**0.021**
≥3 metastatic sites[c]	2.526 (1.344–4.749)	**0.004**	2.705 (1.398–5.234)	**0.003**
Fulvestrant vs letrozole	0.731 (0.446–1.199)	0.214	0.836 (0.509–1.372)	0.479

Each model contains the covariates of the primary models (Table 3) together with endocrine resistance, three or more metastatic sites and the endocrine partner (Enter method); — indicates that the marker was not included in the corresponding model. These extended models were exploratory (9.0 events per variable for overall survival). [a] LAR was modelled on the natural-logarithmic scale,

with the hazard ratio expressed per doubling of LAR. [b] Defined according to the ESO–ESMO ABC criteria as relapse during or within 12 months of completing adjuvant endocrine therapy, or receipt of endocrine therapy for metastatic disease before the CDK4/6 inhibitor. [c] Number of involved sites among bone, lymph node, lung, liver and brain. Bold p-values denote statistical significance ($p < 0.05$). Abbreviations: CDK, cyclin-dependent kinase; CI, confidence interval; GPS, Glasgow Prognostic Score; HR, hazard ratio; LAR, lactate dehydrogenase-to-albumin ratio.

Table S3. Comparison of LAR and GPS on Equal Terms (n = 139).

Comparison	Overall survival (72 events)		Progression-free survival (110 events)	
	**Estimate**	**p**	**Estimate**	**p**
Primary analysis (continuous LAR vs binary GPS)[a]				
AIC, LAR-based / GPS-based model	612.3 / 624.6	—	906.4 / 917.3	—
ΔAIC (GPS − LAR)	12.3	—	10.9	—
C-index, LAR vs GPS	0.609 vs 0.572	—	0.579 vs 0.540	—
ΔC-index (LAR − GPS)	0.037	>0.20	0.039	>0.20
Binary form				
LAR > 46.27 vs ≤ 46.27, adjusted HR (95% CI)	2.19 (1.28–3.74)	**0.004**	1.54 (1.02–2.33)	**0.039**
GPS ≥1 vs 0, adjusted HR (95% CI)	1.72 (1.05–2.82)	**0.032**	1.32 (0.88–1.98)	0.187
AIC, LAR-based / GPS-based model	620.1 / 624.6	—	914.6 / 917.3	—
ΔAIC (GPS − LAR)	4.5	—	2.7	—
C-index (95% CI), LAR	0.581 (0.519–0.642)	—	0.554 (0.502–0.606)	—
C-index (95% CI), GPS	0.572 (0.510–0.633)	—	0.540 (0.489–0.590)	—
ΔC-index (LAR − GPS)[b]	0.010	0.754	0.014	0.616
Three-level form				
LAR tertile 2 vs 1, adjusted HR (95% CI)	1.80 (0.96–3.39)	0.068	1.21 (0.74–1.96)	0.446
LAR tertile 3 vs 1, adjusted HR (95% CI)	2.41 (1.28–4.51)	**0.006**	1.65 (1.02–2.67)	**0.043**
GPS 1 vs 0, adjusted HR (95% CI)	1.61 (0.95–2.74)	0.077	1.26 (0.82–1.94)	0.298
GPS 2 vs 0, adjusted HR (95% CI)[c]	2.39 (0.91–6.27)	0.076	1.74 (0.73–4.12)	0.211
AIC, LAR-based / GPS-based model	622.9 / 626.1	—	916.8 / 918.9	—
ΔAIC (GPS − LAR)	3.2	—	2.0	—
C-index (95% CI), LAR	0.579 (0.511–0.653)	—	0.548 (0.491–0.605)	—
C-index (95% CI), GPS	0.576 (0.519–0.639)	—	0.543 (0.492–0.596)	—
ΔC-index (LAR − GPS)[b]	0.003	0.963	0.005	0.912

Each index was entered into a separate multivariable Cox model adjusted for lymph-node metastasis, visceral metastasis, Ki-67 ≥50% and the line of CDK4/6 inhibitor therapy (Enter method). In the binary form, LAR was dichotomised at the prespecified threshold of 46.27; in the three-level form, LAR was categorised into tertiles of the present cohort (22.14–44.62, 44.65–57.21 and 58.16–337.73) and GPS into its three original categories (0, n = 95; 1, n = 38; 2, n = 6). [a] Reproduced from Tables 3 and 4 for comparison; LAR was modelled on the natural-logarithmic scale. [b] Paired bootstrap

comparison of Harrell's concordance indices (2000 resamples). [c] Based on six patients; the estimate is unstable. AIC = −2 log-likelihood + 2k, where k is the number of model parameters; a lower AIC indicates better model fit. Bold p-values denote statistical significance ($p < 0.05$). Abbreviations: AIC, Akaike information criterion; CDK, cyclin-dependent kinase; CI, confidence interval; GPS, Glasgow Prognostic Score; HR, hazard ratio; LAR, lactate dehydrogenase-to-albumin ratio.
